# Clinical Disorders in Cystic Fibrosis That Affect Emergency Procedures—A Case Report and Review

**DOI:** 10.3390/jcm14093187

**Published:** 2025-05-05

**Authors:** Sylwia Jarzynka, Mateusz Dobrosz, Sebastian Jaworski, Kamil Jóźwicki, Sebastian Wierzba, Olga Barbarska, Anna Minkiewicz-Zochniak

**Affiliations:** 1 Department of Medical Biology, Medical University of Warsaw, Litewska 14/16, 00-575 Warsaw, Poland; sylwia.jarzynka@wum.edu.pl; 2 Student Scientific Club Agar, Department of Medical Biology, Medical University of Warsaw, Litewska 14/16, 00-575 Warsaw, Poland; mateuszdobrosz@outlook.com (M.D.); skjaworsky@gmail.com (S.J.); jozwicki.kamil98@gmail.com (K.J.);; 3 Emergency Department, Pediatric Teaching Clinical Hospital, University Clinical Centre of the Medical University of Warsaw, Zwirki and Wigury 63A, 02-091 Warsaw, Poland; 4 Medical Emergency Department, Medical University of Warsaw, Litewska 14/16, 00-575 Warsaw, Poland; 5 School of Medical & Health Sciences, University of Economics and Human Sciences in Warsaw, Okopowa 59, 01-043 Warsaw, Poland; o.barbarska@gmail.com

**Keywords:** genetically determined diseases, cystic fibrosis, emergency medical services, paramedics, pre-hospital rescue

## Abstract

Cystic fibrosis (CF) is a multisystemic disease caused by a genetic defect, namely a mutation in the CFTR gene, that results in the production of an abnormal protein that regulates the flow of chloride ions through epithelial cells, leading to the dehydration of secreted mucus and changes in its biological properties. Chronic inflammation and recurrent respiratory infections progressively damage lung tissue, leading to respiratory and cardiorespiratory failure. This study aims to present a clinical case and explore the clinical changes in CF that may influence the provision of pre-hospital first aid. The study presents a case report of a 23-year-old CF patient undergoing evaluation for lung transplantation, infected with *Pseudomonas aeruginosa* and *Staphylococcus aureus* with the MSSA phenotype, and in a severe condition due to infectious exacerbation. Despite antibiotic treatment, the patient’s condition deteriorated, leading to respiratory failure and cardiac arrest. Emergency measures were taken to maintain airway patency—the patient was sedated, intubated, and connected to a ventilator. CF involves systemic complications that, during exacerbations, may require urgent interventions. Cystic fibrosis is associated with multiple systemic complications, some of which may, during exacerbations, require emergency medical interventions. Providing care to this patient group involves specific procedures addressing the consequences of the underlying disease. Due to increasing survival rates and the emergence of new phenotypes, there is a need for the continuous education of medical personnel, including emergency responders, regarding the management of genetically determined diseases. This study underscores the importance of recognizing CF’s complex nature and adapting emergency care accordingly to ensure timely and effective intervention in life-threatening situations.

## 1. Introduction

Cystic fibrosis is a multisystemic genetically determined disease. Its occurrence is determined by a genetic defect, namely a mutation in the CFTR gene (cystic fibrosis transmembrane conductance regulator) located on the long arm of chromosome 7 (7q31.2), that results in the production of an abnormal protein that regulates the flow of chloride ions through epithelial cells [1,2]. The faulty structure of the protein causes the mucus produced by the organs affected by cystic fibrosis to be thicker and stickier than that of healthy individuals. The disease is inherited in an autosomal recessive manner, and the severity of symptoms depends on the specific mutation present in the gene. Over two thousand mutations have been identified within the CFTR gene, with approximately seven hundred known to cause disease [2,3,4]. Cystic fibrosis affects about 56,144 individuals in Europe (registered across 42 countries) and 30,000 individuals in the US, respectively, and approximately 89,000 individuals worldwide, according to registries [5,6]. The European Cystic Fibrosis Society conducted a quantitative estimation regarding the number of individuals undergoing treatment for cystic fibrosis in Europe. The findings indicated that in Poland in 2022, the prevalence of CF ranged from 1620 registered patients not lost to follow-up to 1524 patients seen [6]. According to a Polish Ministry report, due to the progressive nature of the condition, the annual incidence of newly diagnosed cases falls within the range of 70 to 80 individuals [7].

Recent studies have shown that changes in the viscoelastic properties of airway mucus play a key role in both acute and long-term disease progression. Specifically, increased mucus elasticity and viscosity correlate with a reduced forced expiratory volume in one second, reflecting impaired mucociliary clearance and persistent inflammation [8].

Moreover, advances in noninvasive monitoring, such as the use of portable low-field nuclear magnetic resonance imaging, allow clinicians to assess the hydration and microstructure of airway mucus through the measurement of the spin–spin relaxation time. These biophysical markers are now being correlated with clinical indicators of airway clearance and ventilation efficiency, offering a promising tool for disease monitoring and therapeutic guidance [9].

The real clinical challenge is the appropriate medical care of CF patients. Thanks to advancements in therapeutic procedures, an increasingly higher survival rate is observed in this disease. Currently, there are significantly more adults with CF; in some European countries, the survival rate reaches even beyond 80 years (e.g., Denmark, United Kingdom, France, and Spain [6]). Additionally, modern diagnostic methods allow for the detection of new phenotypes of the disease, associated with mutations other than just the commonly occurring F508del mutation. These new phenotypes vary in symptoms and time of diagnosis. Therefore, there are still patients in the population who are diagnosed as CF-positive late, even in adulthood. There is also a group of people who remain undiagnosed due to mild symptoms.

In the future, this situation may change, as since 2019, screening tests for CF have been performed in newborns in Poland. The prevalence of this multisystemic disease continues to rise, and it is essential that doctors and paramedical staff are trained to appreciate its complex nature in adults, and that adequate resources are allocated to ensure proper standards of patient care.

Paramedics are often the first medical responders performing rescue operations for CF patients during disease exacerbation. Their actions directly affect the survival of CF patients. This task is particularly challenging when treating small babies or undiagnosed patients. Some of the treatment procedures for these patients resemble those for chronic obstructive pulmonary disease (COPD) patients, but there remain specific differences in emergency interventions in relation to the patient’s condition and multi-organ atypical clinical symptoms [10].

This study aims to present a case study of an adult patient with cystic fibrosis and to outline the principles of emergency pre-hospital care in life-threatening situations, considering the specific clinical manifestations that may impact rescue procedures. Cystic fibrosis patients require life-saving interventions many times throughout their lives. This study was prepared based on a clinical case description and a review of the available scientific literature, with the novelty of the work lying in the integration of a detailed pre-hospital emergency case report with a narrative review of current clinical and pathophysiological concepts in cystic fibrosis. To our knowledge, such a combined approach remains rare in the literature and aims to offer practical guidance for emergency responders and healthcare professionals.

## 2. Cystic Fibrosis Case Report

### 2.1. Emergency Intervention in Critical Condition

At the age of twenty-three, the patient was undergoing evaluation for lung transplantation. Unfortunately, he suddenly developed signs of a severe infection and required immediate emergency medical care. The patient had respiratory and circulatory failure. Respiratory and cardiac arrest occurred. Life-saving measures were undertaken to maintain airway patency: the patient was sedated and intubated with a 7 mm endotracheal tube, and mechanical ventilation was initiated. His symptoms of a severe shortness of breath, cyanosis, acidosis and difficulty expectorating purulent secretions worsened. The patients’ oxygen saturation on ambient air dropped to 54%.

During hospitalization in the regional hospital, an exacerbation of an infectious process with a multifactorial course involving *Pseudomonas aeruginosa* and MSSA (methicillin-sensitive *Staphylococcus aureus*) was diagnosed. Despite the initiation of empirical treatment with ciprofloxacin and cefadroxil, the patient’s condition continued to worsen. The administered therapy may have been suboptimal. Most strains of *S. aureus* and *P. aeruginosa* are susceptible to ciprofloxacin at increased exposure. In the context of polymicrobial exacerbations, current guidelines recommend the use of broad-spectrum therapies, aminoglycosides, beta-lactams, macrolides, glycopeptides or polymyxins, depending on local resistance patterns and individual patient history [11].

The patient was urgently transferred to the provincial hospital with a cystic fibrosis treatment unit. Upon admission, the patient was in critical condition—unconscious, intubated, and mechanically ventilated. Cyanosis was observed on the distal parts of the body, along with edema in the lower extremities. The controlled respiratory rate was twenty-four breaths per minute, his heart rate was 110 beats per minute, and his blood pressure was 110/70 mmHg. The patient’s skin was pale, and he appeared markedly emaciated, with significantly reduced subcutaneous fat tissue, consistent with severe malnutrition. On physical examination, the abdomen was soft and level with the thoracic cage. Imaging studies revealed multiple overlapping patchy and streaky opacities in the lungs, with no significant changes compared to prior X-rays.

An echocardiogram performed in the subxiphoid view (due to thoracic anatomy and emphysema) showed signs suggestive of pulmonary hypertension, right ventricular overload, and borderline contractility. Laboratory findings revealed anemia with anisocytosis and reduced hemoglobin and hematocrit values in the red blood cell system. In the white blood cell system, leukocytosis, monocytosis, neutrophilia, and lymphocytopenia were observed. Biochemical tests showed elevated C-reactive protein levels, while other parameters remained within normal limits. The liver was not enlarged, and the spleen was not palpable. On auscultation, wheezing and crackles were heard; these were more pronounced on the left side, particularly in the upper fields. No pleural rub was detected, and percussion resonance was normal. The skeletal system showed no significant abnormalities, and the patient exhibited clubbing of the fingers.

To improve the patient’s overall condition, treatment targeting the inflammatory state was initiated. Antipseudomonal and antistaphylococcal antibiotic therapy, including meropenem, colistin, and cloxacillin, was administered. Intravenous anti-inflammatory therapy included methylprednisolone. During the subsequent hospitalization period, after the patient’s condition stabilized and extubation was performed, non-invasive ventilation was introduced to provide respiratory support without the need for reintubation, using a face mask to deliver positive airway pressure.

To improve pulmonary gas exchange and liquefy thick, purulent secretions in the bronchial tree, nebulized therapies with colistin, salbutamol, formoterol, and dornase alfa were used. Edema was managed with furosemide. Additional supportive therapies included pancrelipase calcium carbonate, vitamin D3, Vitamin E, and potassium chloride. A gradual improvement in the patient’s condition was observed, corroborated by laboratory test results.

Due to the need to improve the patient’s nutritional status (weight at admission was 38 kg, height 165 cm, BMI 14), which was a prerequisite for lung transplantation, a percutaneous endoscopic gastrostomy (PEG) was placed, and parenteral nutrition with industrial formula Nutrison Energi (Nutricia, Poland) was initiated. Imaging studies at the Institute of Tuberculosis and Lung Diseases showed no abnormalities in the liver, pancreas, or kidneys, which were of normal size and structure. Minor ascites was observed. Further evaluation of the hepatobiliary system was warranted due to imaging findings suggestive of liver cirrhosis. The condition of the gallbladder also required further evaluation. No abnormalities were noted in a soft tissue ultrasound or abdominal X-ray.

After completing treatment, the patient was discharged with a final diagnosis of chronic respiratory failure due to cystic fibrosis. Discharge recommendations included the systematic use of inhaled mucolytics, pancrelipase with meals, and supplementation with vitamins D, E, and K. With the PEG in place, the patient was instructed to report to gastroenterology outpatient care for monitoring and potential replacement, and to the Thoracic Surgery and Transplantology Department in Szczecin to assess his qualification for lung transplantation. The patient also qualified for home oxygen therapy.

### 2.2. Previous Medical History of a Patient

**Figure 1:**
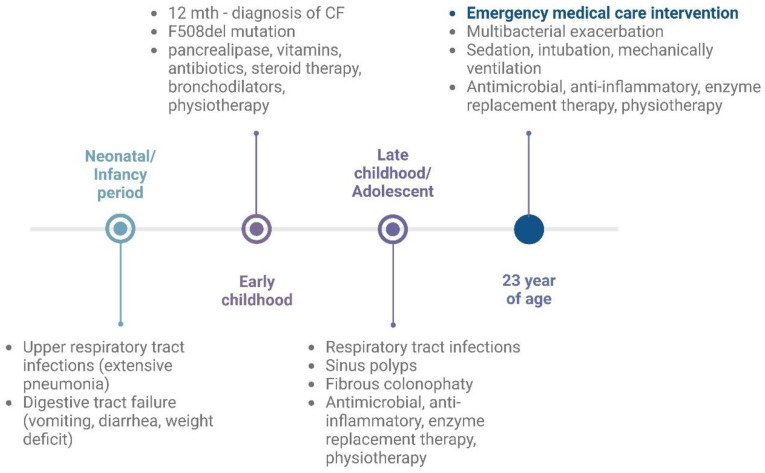
Cystic fibrosis case report—medical history timeline. Created in https://BioRender.com, (accessed on 22 January 2025).

The medical history timeline of the case study is presented in Figure 1.

**Neonatal Period.** The child was born at 39 weeks of gestation via vaginal delivery. The boy was evaluated at 10 points on the Apgar scale. Neonatal parameters: birth weight—3650 g, length—56 cm, head circumference—38 cm, chest circumference—36 cm. The stay in the neonatal ward was uneventful. On the third day, the child was discharged home.

**Infancy Period.** At 2 months of age, the child began to experience recurrent upper respiratory tract infections manifesting as a runny nose and cough, without signs of airway obstruction. Due to chronic infections, the child was treated in an outpatient setting. Until the age of 3 months, the child was breastfed and gained weight appropriately. From 4 months of age, the boy was formula-fed with Bebiko 1 (Nutricia, Warsaw, Poland). By 4 months, weight gain ceased, and the child began experiencing regurgitation after feeding and mucus in the stool. The pediatrician treating the child suspected milk intolerance. Due to the chronic symptoms of respiratory tract infections, at 5 months of age, the child was transitioned to a milk substitute formula, Humana MCT (Humana, Bremen, Germany), a diet specifically designed with a balanced fat and carbohydrate content for children with digestive and absorption disorders. However, no clinical improvement was observed.

By 5 months, persistent symptoms suggestive of chronic respiratory infection prompted a change in diet to accommodate digestive issues, yet no improvement was noted. At 10 months, the child developed diarrhea and vomiting, resulting in hospitalization due to a severe decline in general health and the onset of respiratory and circulatory failure caused by extensive pneumonia. The child was found to have a significant weight deficit. The predominant symptoms included a moist, ineffective cough, an oxygen saturation of 87%, and cyanosis in the distal parts of the body. The child was severely malnourished, with a diagnosed weight deficit of 45%. Physical examination revealed a distended abdomen and an enlarged liver extending to the umbilical line. Fatty stools were also detected.

After a three-week stay in the Pediatric Ward and intensive pharmacological treatment, a gradual improvement in the child’s clinical condition was observed, with restored respiratory and circulatory efficiency. Saturation improved to 94–97%. Due to a persistent low body weight (6170 g), the child was referred for further treatment to the Gastroenterology Department at the Institute of Mother and Child in Warsaw.

**Early Childhood**. At 12 months of age, cystic fibrosis was diagnosed based on laboratory test results at the Gastroenterology and Nutrition Clinic of the Institute of Mother and Child in Warsaw. The child’s sweat chloride level was 125.7 mg/L. After a one-month hospitalization in the Gastroenterology Department, celiac disease was ruled out. Subsequent treatment continued at the Pediatric Clinic of the Institute of Mother and Child in Warsaw. The sweat test was repeated three times, with positive results. A genetic DNA test revealed mutations in both alleles of the CFTR gene, confirming the F508del genotype for cystic fibrosis. The child continued to exhibit weight and growth deficits, falling below the 3rd percentile (weight 6850 g, height 70 cm). Targeted treatment was introduced, including a high-calorie diet, the enzyme preparation pancrelipase, vitamins, antibiotics, steroid therapy, and bronchodilators. Physiotherapy was also initiated, leading to a significant improvement in the patient’s health. The boy remained under the care of the Cystic Fibrosis Clinic at the Institute of Mother and Child in Warsaw.

**Late Childhood and Adolescence**. During school years, the patient underwent multiple surgeries for recurrent nasal cavity and sinus polyps, likely due to sinusitis. Chronic constipation and fibrosis colonopathy were confirmed. As a teenager, the patient was hospitalized in Piaseczno Hospital, where fibrosing colonopathy was identified. The patient was treated for one year with sulfasalazine, which has antibacterial, anti-inflammatory, and immunosuppressive properties, to regulate bacterial colonization of the gastrointestinal tract.

## 3. Narrative Review

### 3.1. Clinical Manifestation of Cystic Fibrosis

The malfunction of the chloride channel leads to a disturbance in the secretion of chloride ions into the extracellular space and the retention of sodium within the cells, which results in dehydration of the secreted mucus and a change in its biological properties. It becomes thick and sticky, and difficult to evacuate naturally. The CFTR protein is present in various organs, including the respiratory system, digestive tract, liver, pancreas, intestines, bones, kidneys, and the male reproductive system (Supplementary Table S1 compiled from sources [8,12,13,14,15,16]) [17,18,19]. Symptoms of cystic fibrosis affecting the respiratory system occur in over 90% of patients. In the respiratory tract, impairment of this protein’s function leads to an increase in mucus thickness, which, when not removed by the mucociliary transport system, results in difficulty evacuating it naturally [20,21]. In the airways, due to the accumulation of secretions and recurrent bacterial infections, a chronic inflammatory state develops, leading to the destruction of bronchial walls and fibrosis surrounding the lung parenchyma [22].

Typically, there is chronic bacterial colonization by pathogens such as *Haemophilus influenzae* type b (Hib serotype) [23], *Staphylococcus aureus* and *Pseudomonas aeruginosa*, which, after transformation into a mucoid subtype, becomes practically impossible to eradicate. Its presence exacerbates bronchial inflammation and destruction [24]. Nevertheless, the most frequently encountered fungi linked to respiratory tract infections in CF patients are *Aspergillus fumigatus* and *Candida albicans*. A very rapid progression of the disease is caused by infection with the antibiotic-resistant bacterium *Burkholderia cepacia* complex (BCC).

Although *Pseudomonas aeruginosa* and BCC bacterial complexes are most associated with severe infections and a worsened prognosis in cystic fibrosis patients, recent studies indicate an increasing prevalence of methicillin-resistant *Staphylococcus aureus* (MRSA) in samples from these patients [25,26,27]. MRSA infections are more frequently observed in young adults after many years of antibiotic therapy compared to children [25]. CF patients colonized with MRSA also have higher hospitalization rates and a greater history of oral, inhaled, and intravenous antibiotic use compared to those who are MRSA-negative patients [25].

It is currently estimated that the prevalence of MRSA detection in the respiratory tracts of CF patients ranges from 20% to as high as 30% [27,28]. Additionally, some MRSA strains contain virulent factors that may enhance their ability to damage infected respiratory tissues. In advanced stages, extensive damage to the lung parenchyma occurs. Areas of fibrosis and emphysema develop, eventually leading to complete respiratory failure [25,26,29].

Gastrointestinal symptoms occur in most CF patients [30]. The pancreas is among the organs most frequently affected at an early stage in individuals diagnosed with cystic fibrosis. In the pancreas, thick, stagnant pancreatic juice blocks the ducts, resulting in their cystic dilation. The activation of proteolytic enzymes promotes chronic inflammation and cell necrosis. This results in exocrine pancreatic insufficiency and, in some cases, endocrine insufficiency. There are also disruptions in the bile ducts, which can lead to fatty liver and cirrhosis (Supplementary Table S1) [30,31]. Thick mucus accumulation in the epididymis and vas deferens during fetal development leads to their obstruction and closure, resulting in azoospermia and infertility in approximately 98% of affected men. In women, the presence of thick cervical mucus creates unfavorable conditions for fertilization. Infertility among women afflicted with cystic fibrosis is less prevalent than in men with the condition—approximately 35–50% of women with cystic fibrosis report experiencing infertility or subfertility. Additionally, around 97% to 98% of men with CF experience infertility due to the abnormal development of the vas deferens, epididymis, and seminal vesicles. Azoospermia arises from the presence of thick secretions in the vas deferens, and approximately 50% of sperm exhibit morphological abnormalities (Supplementary Table S1) [32,33].

The main clinical problem in cystic fibrosis is respiratory system disorders. Chronic inflammation and frequent recurrent respiratory infections lead to lung tissue damage and the development of respiratory distress syndrome and progressive respiratory failure. Bacterial respiratory infections persist as a determining factor in the clinical trajectory and prognostication of patients with cystic fibrosis [34]. As the disease progresses, exercise tolerance declines, followed by the appearance of resting dyspnea. Massive bronchiectasis can lead to hemoptysis, and the presence of bullae promotes the development of pneumothorax. Over time, cystic fibrosis leads to complete respiratory failure, right-sided heart failure, and death. In the end stage of the disease, patients require home oxygen therapy and hospital care [35]. The secretion of pancreatic enzymes responsible for the breakdown of fats, proteins, and carbohydrates is also impaired. As a result, the body does not absorb many essential nutrients, and the patient becomes malnourished and underweight.

One of the procedures able to prolong the life of patients with cystic fibrosis is lung transplantation (LTx). For many people, lung transplantation remains the only effective treatment method. Patients who have already used all treatment methods and who have no significant contraindications to this form of therapy may be qualified for transplantation. However, the timing of transplantation is crucial for success, especially if the simultaneous transplantation of abdominal organs, such as the liver, is considered. While multi-organ recipients show a higher mortality rate in the first year after transplantation, there was no significant difference in long-term survival compared to patients with LTx or liver transplants alone. The median survival of patients with cystic fibrosis after lung transplantation is 8.3 years. We estimate that in Poland, over the last 11 years, a total of over 60 patients with CF have received lung transplants [36,37,38].

Currently, CFTR modulators have significantly reduced the number of lung transplants in patients with CF. Accumulating evidence indicates that CFTR modulators represent a paradigm-shifting therapeutic breakthrough in cystic fibrosis, rather than merely one of several available treatment options [39]. These agents have demonstrated clinically meaningful improvements in pulmonary function, reductions in exacerbation rates, enhanced nutritional status, and a better health-related quality of life—even among individuals with advanced lung disease. These findings support the classification of CFTR modulators as essential disease-modifying therapies within the framework of contemporary cystic fibrosis management [40,41].

A limited number of patients with CF have CFTR mutations that are eligible for ivacaftor; approximately 40% to 50% can be treated with dual combination therapy (lumacaftor–ivacaftor or tezacaftor–ivacaftor). However, the greatest clinical benefit has been seen with the triple combination therapy of elexacaftor–tezacaftor–ivacaftor, developed for patients with at least one F508del CFTR allele (representing 80% to 85% of patients with CF) [42]. This therapy significantly improves lung function, reduces the number of exacerbations, and improves the patient’s overall condition. In Poland, since 1 April 2025, CFTR modulators Kaftrio + Kalydeco (ivacaftor + tezacaftor + elexacaftor and ivacaftor) have been used as drugs for cystic fibrosis patients from 2 years of age.

### 3.2. Cardiovascular Impact

Right ventricular failure, commonly associated with pulmonary hypertension in cystic fibrosis, emerges as a critical life-threatening condition. Chronic hypoxemia and the progressive destruction of the lung parenchyma result in increased pulmonary vascular resistance, leading to hypertrophy and the subsequent dysfunction of the right ventricle. Elevated systolic pulmonary artery pressure can persist even in the absence of overt pulmonary hypertension, gradually impairing contractility and diastolic properties. Similarly, left ventricular dysfunction, encompassing both systolic and diastolic abnormalities, poses a severe risk.

Myocardial fibrosis and necrosis, attributed to deficiencies in essential vitamins and the aberrant activation of pancreatic enzymes, compromise ventricular compliance and function. The hypertrophic remodeling of the left ventricle (LV), exacerbated by elevated levels of neurohormonal mediators such as aldosterone and angiotensin II, further limits myocardial reserve. Additionally, arrhythmias, often associated with myocardial fibrosis and CFTR-related ion transport abnormalities, can lead to sudden cardiac arrest. Pulmonary hypertension, whether overt or subclinical, exacerbates these conditions, increasing the afterload on the right ventricle (RV) and contributing to the development of cor pulmonale. Collectively, these pathophysiological processes underscore the critical role of vigilant cardiovascular monitoring and early intervention in CF patients.

### 3.3. Emergency Evaluation and Procedures for Patients with Cystic Fibrosis

Patients with cystic fibrosis may require appropriate medical emergency procedures in pre-hospital settings, provided by the Emergency Medical Team, depending on the stage of cystic fibrosis progression [43]. Symptoms typically involve multiple organ systems, primarily the respiratory and digestive systems [1].

The ABCDE approach is a systematic examination used by healthcare practitioners to assess and manage patients in emergencies, prioritizing life-threatening conditions using the ABCDE acronym (Airway, Breathing, Circulation, Disability, Exposure). For patients with CF, who frequently experience acute exacerbations, this approach is crucial due to the disease’s chronic and complex nature. The ABCDE framework ensures airway clearance and adequate ventilation, which are vital for CF patients suffering from mucus obstruction and reduced lung function [44]. Each stage of this examination is extremely important and must not be skipped.

The patient’s history is obtained using the SAMPLE mnemonic (Symptoms, Allergies, Medications, Past medical history, Last oral intake, and Events leading up to the present illness), which is a structured approach used in medical assessments to gather comprehensive patient information.

The management of CF requires a multifaceted approach, where airway clearance techniques (ACTs) and physical activity play key roles in preserving lung function and improving quality of life. While conventional chest physiotherapy remains the gold standard, alternative ACTs provide effective, self-administered options that may improve patient adherence. Exercise is increasingly recognized as a valuable adjunct to traditional ACTs, promoting deep breathing and effective coughing. Furthermore, the integration of digital health technologies is advancing remote monitoring and personalized treatment [45,46].

The initiation and duration of intravenous (IV) antibiotic therapy for CF exacerbations should be personalized based on the patient’s response, aiming to achieve optimal pharmacokinetic and pharmacodynamic targets. Current evidence supports early initiation, with the treatment duration typically extending from 7 to 21 days, and being tailored to the clinical response. Early responders may benefit from shorter courses (7–10 days), while late responders, particularly hospitalized patients, may require longer treatment (14–21 days). This individualized approach prioritizes treatment efficacy over rigid durations, aiming to control exacerbations, minimize lung damage, and optimize health outcomes [47,48]. Mechanical ventilation is recommended for patients with reversible causes of acute respiratory failure who are otherwise in good condition, as well as for those awaiting lung transplantation [49,50].

#### 3.3.1. Respiratory Tract

The most serious complication of cystic fibrosis is respiratory failure, a condition characterized by impaired respiratory system function, leading to progressive hypoxemia, which ultimately prevents adequate tissue oxygenation [51]. There are two types of respiratory failure: type I (hypoxemic) and type II (hypoxemic-hypercapnic). Type I respiratory failure occurs when the respiratory system is unable to deliver sufficient oxygen to the body, resulting in hypoxemia. Type II respiratory failure occurs when the respiratory system fails to adequately eliminate carbon dioxide from the body, leading to hypercapnia [52].

Respiratory failure due to the exacerbation of cystic fibrosis can develop due to the insufficient ventilation of the lung alveoli. Chronic alveolar hypoxia, progressive pulmonary vascular structural damage, and a sustained elevation in cardiac minute volume may culminate in the development of pulmonary hypertension and pulmonary fibrosis [22]. Typical symptoms include a shortness of breath, a worsening cough, chest pain, and hemoptysis, especially in older patients with advanced bronchopulmonary disease. Hemoptysis in CF results from chronic infection and inflammation of the airways, contributing to hypertrophy of the bronchial arteries and the formation of granulation tissue, making the vessels prone to damage during coughing [53,54].

It should be noted that a shortness of breath is a subjective sensation and may not correspond to pulse oximetry values. Clinically, signs of hypoxemia can be observed, including the exacerbation of underlying case cystic fibrosis symptoms, sometimes accompanied by an increase in the work of accessory respiratory muscles and paradoxical movements of the chest [22,52]. Hypoxia not only serves as a symptom of advanced CF lung disease but may also act as a causative factor in worsening the disease’s severity. Symptoms of hypoxemia may include:

- Cyanosis is a change in the color of the skin due to an increased concentration of deoxyhemoglobin >5 g/dL. Central cyanosis is observed around the mouth, mucous membranes of the oral cavity and tongue. Peripheral cyanosis is observed in the distal parts of the limbs. Differential cyanosis presents as a bluish discoloration between the upper and lower extremities [55];
- Pulmonary hypertension—hypoxia can lead to increased pulmonary artery pressure, contributing to pulmonary hypertension, which is a common complication in CF patients [56];
- Reduced lung function—hypoxia leads to a decreased forced expiratory volume and forced vital capacity in CF patients [56];
- Inflammation and infection—hypoxia worsens inflammation and promotes the growth of pathogens like *Pseudomonas aeruginosa* in CF lung infections [57];
- Anxiety, agitation, fear, or a loss of consciousness are signs of central nervous system hypoxia;
- Metabolic acidosis caused by hypercapnia is a sign of anaerobic metabolism in the tissues. It occurs in type II respiratory failure.

In patients with cystic fibrosis and acute respiratory failure, it is not advisable to aim for saturation levels of 94–98%. The target oxygen saturation (SaO_2_) in this group of patients is 88–92% [58]. It should be remembered that the excessive inhalation of oxygen can lead to hypoventilation due to a decrease in peripheral chemoreceptor stimulation, which subsequently leads to an increase in the partial pressure of carbon dioxide (PaCO_2_) [58,59,60,61].

Active oxygen therapy is a method of mechanically delivering oxygen or a breathing mixture into the airways and lung alveoli using assisted breathing. It is necessary in cases of insufficient natural respiratory drive and involves the use of both instrument-assisted and non-instrument-assisted airway clearance techniques. Additionally, a self-inflating bag with reservoir allows for achieving very high concentrations of oxygen in the breathing mixture. It is worth emphasizing that in the Polish State Emergency Medical System, intubation performed by a paramedic or nurse is only possible in cases of sudden cardiac arrest [62,63].

**Figure 2:**
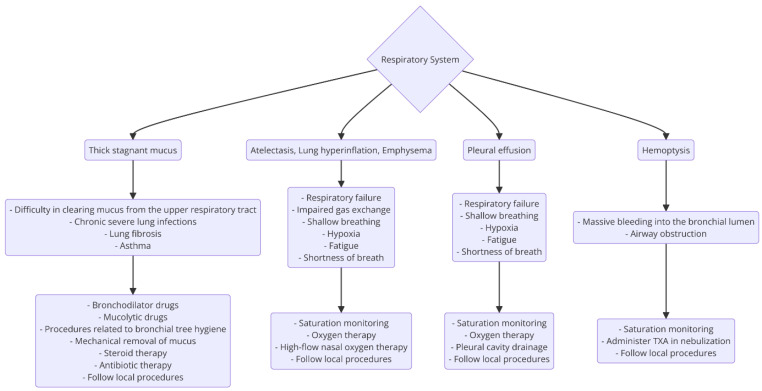
Disorders in the respiratory system related to cystic fibrosis impact the process of emergency medical interventions. Source: self-prepared based on [17,18,19,64,65,66].

Significant respiratory complications of CF also include the occurrence of pneumothorax. The frequency of pneumothorax increases with the age of CF patients. The cause of pneumothorax is usually the rupture of a subpleural emphysematous bulla due to significant damage to the lung parenchyma. As noted above, cystic fibrosis also has clinical manifestations beyond the respiratory system, particularly in the digestive system. Based on a clinical case, the most important disorders and potential life-saving medical interventions are presented in Figure 2.

The emergency management of acute respiratory failure is based on identifying the cause and treating it accordingly. The most important symptomatic treatment is oxygen therapy [67]. There are two types of oxygen therapy: passive and active. Passive oxygen therapy is administered while the patient maintains sufficient ventilation. Masks and nasal oxygen catheters are used. The possible concentrations of inhaled oxygen and types of equipment used for passive oxygen therapy in prehospital care are presented in Table 1. There are no precise guidelines for oxygen masks used specifically in CF patients. The masks recommended for COPD patients may be most appropriate for use in CF as well. However, a patient in an emergency condition can be subjected to inhalation using available sets, so it is worth summarizing them in Table 1.

When providing medical emergency care to individuals with cystic fibrosis, it is crucial to remember the specific symptoms of this condition as well as the mechanisms that lead to their occurrence. Adhering to the principles of standardization and using therapeutic methods with scientifically proven efficacy, it is worth emphasizing that the primary effective medications in treating patients with cystic fibrosis are hypertonic saline solution and dornase alfa [54]. Nebulization using a 3–7% sodium chloride solution is a safe, cost-effective, and efficient therapeutic method. Regular inhalations, performed 2–4 times daily with a dose of 4–5 mL per session, significantly reduce the frequency of bronchial and pulmonary exacerbations, as well as inflammatory states. The mechanism of action is based on enhancing mucociliary clearance and facilitating the removal of secretions. To minimize the risk of bronchospasm, it is recommended that inhalation is conducted after the prior administration of bronchodilators, followed by appropriately tailored physiotherapy exercises. Due to its low invasiveness, therapy with a hypertonic saline solution is well-tolerated by both children and adult patients [1,11].

Dornase alfa is a synthetic, glycosylated, and phosphorylated human deoxyribonuclease protein that hydrolyzes the extracellular DNA present in mucus, leading to a significant reduction in the viscosity of secretions. In sputum, decaying leukocytes leave large amounts of extracellular DNA in the form of fibrous polyanions, which increase the viscosity of mucus. The regular administration of dornase alfa improves the overall condition of the patient and significantly reduces the frequency of exacerbations in the bronchi and lungs [54,68].

There have been documented cases of using N-acetylcysteine (NAC) in nebulized form for patients in life-threatening conditions when standard treatment methods have proven ineffective. The administration of NAC resulted in the breakdown of mucus plugs obstructing effective ventilation. Currently, no adequate clinical studies have been conducted to confirm the efficacy of NAC in emergency medicine [69]. In cases of bronchial obstruction in some patients, beta2-antagonist drugs are used. Their mechanism of action involves activating beta2-adrenergic receptors located in the bronchi, leading to airway dilation and reduced muscle tension in their walls. At standard doses, these medications act selectively on beta2 receptors; however, at higher doses, they may induce tachycardia by stimulating beta1 receptors present in the heart muscle [54,70].

When a patient develops cardiopulmonary failure due to hemoptysis, it should be assumed that massive hemoptysis is present. This is a life-threatening and health-critical condition. Despite the lack of detailed studies on massive hemoptysis, the use of tranexamic acid (TXA) in nebulized form is recommended. Due to its safety profile, it is advised that nebulization is administered every 8 h with a dose of 500 mg of TXA [71].

**Table 1:** Oxygen concentrations in the respiratory mixture when using a particular type of oxygen mask/tube [72,73,74].

Type of Oxygen Mask/Tube	The Obtained Values of FiO_2_
Standard nasal cannula	It is widely accepted that 1 L/min = 24% (each increase by 1 L/min, in the range of 1–6 L/min, increases the concentration by about 4%). The precise measurement of FiO_2_ is difficult due to the variation in breathing patterns, including both tidal volume and respiratory rate.
Simple face masks	It is commonly accepted that 40–60% is used at a flow rate of 5–10 L/min, depending on the oxygen flow rate and the patient’s breathing pattern. A low rate of less than 5 L/min is not recommended due to the risk of CO_2_ rebreathing and increasing resistance during inhalation.
Venturi masks	Venturi masks allow for the precise administration of a specific oxygen concentration at the rate specified by the manufacturer (24%, 25%, 28%, 35%, 40%, 50%, 60%). If the patient is breathing at a rate of >30 breaths/min, the oxygen flow should be increased by 50% above the manufacturer’s recommendations.
High-concentration reservoir mask (non-rebreathing mask)	It is generally acknowledged that 60–90% is used at a flow rate of 15 L/min, but FiO_2_ depends on the patient’s breathing pattern and the proper fit of the mask to the face.

#### 3.3.2. Digestive System

**Figure 3:**
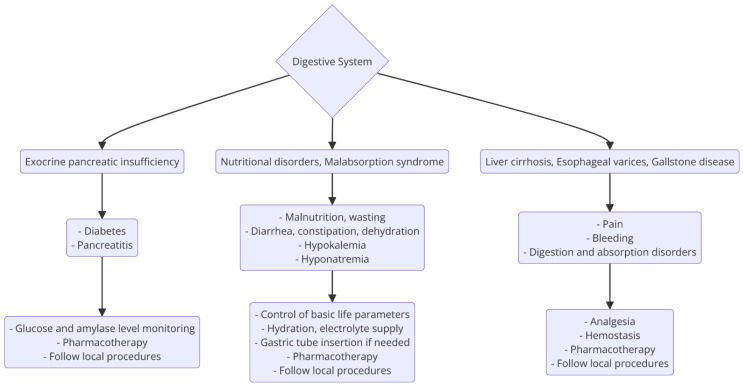
Disorders of the digestive tract related to cystic fibrosis impact the process of emergency medical interventions. Source: self-prepared based on [76,77,78,79,80,81].

Cystic fibrosis significantly affects the digestive system, leading to notable gastrointestinal complications that present challenges in emergency medical scenarios (Figure 3). Key issues include pancreatic insufficiency and malabsorption, resulting in malnutrition that can complicate emergency interventions. This includes Distal Intestinal Obstruction Syndrome, characterized by intestinal blockage due to thickened secretions causing abdominal pain and vomiting, and meconium ileus and constipation, which exacerbate gastrointestinal issues and require precise management to avoid misdiagnosis [65,75].

Additionally, intestinal dysbiosis and inflammation in CF patients impact the gut-lung and gut–liver axes, complicating respiratory and hepatic functions during emergencies. Effective first aid requires emergency responders to be adept at recognizing and managing these symptoms, utilizing specialized equipment for rapid medication delivery and vital sign monitoring, and ensuring robust coordination with healthcare providers through real-time data and remote guidance. Addressing these gastrointestinal complications with a multidisciplinary approach is crucial for enhancing preparedness and improving outcomes in CF-related emergencies [65,75].

#### 3.3.3. Sudden Cardiac Arrest

Sudden cardiac arrest is defined as the sudden cessation of mechanical cardiac function, leading to the cessation of circulation and the secondary cessation of breathing and central nervous system function [82]. There are two types of resuscitation procedures:

○ Basic Life Support (BLS), which consists of chest compressions and rescue breaths (cardiopulmonary resuscitation, CPR), with the use of an automated external defibrillator (AED).
○ Advanced Life Support (ALS), which includes CPR, defibrillation, maintaining airway patency, pharmacotherapy, and all other procedures aimed at restoring spontaneous cardiac action.

In the BLS protocol, the rescuer should use the standard algorithm provided by the European Resuscitation Council in the 2021 Resuscitation Guidelines [66,82]. During ALS management in a patient with cystic fibrosis, it is important to remember that cardiac arrest may result from chronic or acute respiratory failure, and procedures outlined in the ERC Guidelines should be implemented [82].

In addition, airway suctioning should be considered due to the accumulation of mucus that impairs ventilation. It is worth noting that these patients are at an increased risk of hyperthermia, which should be treated already in the pre-hospital phase by aiming to lower the temperature below 39 °C. If hyperthermia accompanies cardiac arrest, standard ALS protocols with continued therapeutic cooling and the use of standard energy doses in defibrillation are applied. Animal studies have shown that hyperthermia reduces positive outcomes compared to normothermia, and the risk of neurological damage increases with every degree above 37 °C [82].

## 4. Discussion

Cystic fibrosis is a systemic disease with diverse clinical expressions, manifesting as chronic bronchopulmonary disease in approximately 90% of cases. This disease is currently incurable and leads to a shortened lifespan in patients, with a progressive decrease in exercise tolerance and eventually the development of resting dyspnea. The condition greatly limits patient’s quality of life due to the necessity of continuous inhalation therapy. Massive bronchial dilatation usually leads to varying degrees of hemoptysis, and the presence of emphysematous bullae promotes the development of pneumothorax. Over time, cystic fibrosis leads to complete respiratory failure, right ventricular heart failure, and ultimately death [43].

Life expectancy is a fundamental indicator used to assess the effectiveness and quality of medical, paramedical, and even family-based pre-hospital care in patients with cystic fibrosis [83,84]. In Poland, the median age at death for individuals with cystic fibrosis is under 24 years—significantly lower and concerning when compared to countries such as Canada, where it reaches approximately 39 years [7].

The number of adult individuals afflicted with cystic fibrosis has been increasing each year, owing to advancements in medical science and the implementation of comprehensive healthcare [84]. Pharmacotherapy, comprising antibiotics, mucolytics, bronchodilators, corticosteroids, and pancreatic enzymes, plays a central role in managing infections and enhancing both respiratory and digestive functions. Adequate hydration, the regular monitoring of blood glucose levels, and adherence to a high-calorie diet are essential components of effective cystic fibrosis management. Treatment strategies, including both hospital and home care, are tailored to overcome challenges such as drug resistance and the need for long-term therapy [85,86]. Antimicrobial stewardship programs are crucial in optimizing antibiotic use and preventing resistance [87,88,89,90].

Physiotherapy is a critical component in the management of CF, including airway clearance methods such as airway clearance techniques [91,92]. The integration of physiotherapy with pharmacological treatments, such as inhaled antibiotics and CFTR modulators, is essential for delivering comprehensive CF care [90,93,94]. Emerging gene therapy approaches, while still under investigation, hold significant promise for long-term treatment by targeting and correcting the underlying genetic mutations responsible for CF [90].

Patients in life-threatening conditions are examined using physical examination and mnemonic assessment techniques (ABCDE, SAMPLE), and in pediatric patients, the Pediatric Assessment Triangle is also used. The primary goal is to minimize the risk of infections, which are often the cause of patient deaths [25,95]. Another important aspect is appropriate clinical and specialized management, as well as timely medical emergency procedures. To limit lung tissue damage, minimally invasive diagnostic and therapeutic methods are employed. Respiratory system disorders are often the most common reason for Emergency Medical Services interventions, hospitalizations, and patient deaths. The target SpO_2_ for this patient group is 88–92% [96].

This study addresses a relevant clinical gap by presenting a detailed real-life case of emergency management in a patient with cystic fibrosis in a pre-hospital setting. The combination of a case report with a narrative literature review provides a novel and practice-oriented perspective that remains underrepresented in current publications. This integrated approach may support more informed and rapid decision-making by emergency teams, particularly in situations where time-sensitive interventions are critical. To the best of our knowledge, this is the first summary to address the topic of rare genetic disorders in relation to rescue procedures. The publication by Sofianopoulos and Williams [97] on the prehospital treatment of patients with CF presenting with hemoptysis based on a retrospective case series from an Australian hospital is noteworthy (lit). The authors emphasize that there are very limited clinical practice guidelines for CF paramedics and that the prompt initiation of treatment is essential. In this context, we believe the manuscript represents a pioneering effort to contextualize complex pathophysiological processes in a manner that is accessible and clinically useful to a broader spectrum of healthcare providers.

Therefore, there is a need to create detailed emergency response guidelines based on current first aid standards for this group of patients. Younger and older children, who are at a particular risk of respiratory failure, represent a very important target group of patients. The topic of pre-hospital emergency procedures in cystic fibrosis patients is extremely important, especially due to the scarcity of publications and guidelines describing this topic. There is a great need for the ongoing education of medical personnel regarding clinical disorders associated with genetically determined diseases [98]. Additionally, the awareness of patients with cystic fibrosis regarding the recognition of exacerbation symptoms is very important. This is crucial because the earlier the patient seeks medical attention, the lower the risk of intubation and severe invasive infection, as was the case in the presented case report.

In conclusion, the clinical case and available literature data described in the study suggest that cystic fibrosis encompasses various disorders that, during exacerbation, may necessitate multiple pre-hospital emergency interventions, emphasizing the importance of preparedness and proactive management strategies. It is essential that doctors, paramedical staff, nurses and physiotherapists are trained to appreciate the complex nature of this multisystemic disease, like other genetically determined conditions, and that adequate resources are allocated to ensure proper standards of patient care. Providing emergency procedures for CF patients primarily requires adapting to the disease’s distinct clinical manifestations, particularly respiratory failure. Given the increased life expectancy of CF patients, the continuous education of medical personnel regarding the spectrum of disorders associated with genetically inherited conditions is imperative, facilitating early recognition and the optimal management of complications in cystic fibrosis patients.

## Data Availability

The original contributions presented in this study are included in the article. Further inquiries can be directed at the corresponding author.

## Supplementary Materials

The following supporting information can be downloaded at: https://www.mdpi.com/article/10.3390/jcm14093187/s1, Table S1. The most common clinical symptoms in patients with cystic fibrosis. Table S1 summarizes data based on previous publications [8,12,13,14,15,16], which are included in the main reference list.
