# Supplementary material for: Clinical Disorders in Cystic Fibrosis That Affect Emergency Procedures—A Case Report and Review
Source: J Clin Med. 2025 May 5;14(9):3187. doi: 10.3390/jcm14093187 (PMC12072408; doi:10.3390/jcm14093187)
Supplement: Supplementary file 1 [file jcm-14-03187-s001.zip › jcm-3526525-supplementary.pdf]

Table S1. The most common clinical symptoms in patients with cystic fibrosis.

Fetal and neonatal	Infancy and post-infancy	Childhood and adulthood
- calcifications in the fetal peritoneal cavity<br>- dilation of the small fetal intestine<br>- meconium ileus detected by ultrasound examination; early cystic fibrosis sign in 20% of newborns; complications: meconium peritonitis, gangrene, intestinal necrosis<br>- pancreatic insufficiency: malabsorption, fatty stools, poor weight gain, growth retardation<br>- prolonged intrahepatic cholestasis	- weight loss<br>- recurrent pneumonia, bronchiolitis<br>- chronic cough<br>- pulmonary exacerbations<br>- early-onset asthma<br>- chronic sinusitis<br>- chronic respiratory tract infections, including those caused by *Pseudomonas aeruginosa* or *Staphylococcus aureus*<br>- chest X-ray abnormalities<br>- recurrent atelectasis, hyperinflation<br>- very salty sweat<br>- dehydration with hypokalemia, hyponatremia, and hypochloremia alkalosis (Bartter ‘s purported syndrome)<br>- collapse during heatwaves<br>- foul-smelling, fatty, bulky stools<br>- symptoms of malabsorption syndrome<br>- prolapse of the rectal mucosa<br>- symptoms of vitamin deficiencies, especially fat-soluble vitamins<br>- hypoprothrombinemia<br>- hypoproteinemia and edema	- chronic rhinosinusitis<br>- recurrent lung infections<br>- chronic respiratory tract infections, including those caused by *Pseudomonas aeruginosa, Staphylococcus aureus, Burkholderia cepacia* complex, *Stenotrophomonas maltophila* or fungal infection<br>- chronic cough<br>- chronic inflammation of the sinuses<br>- nasal polyps<br>- bronchiectasis<br>- hemoptysis<br>- obstructive sleep apnea and nocturnal hypoxemia<br>- clubbing of fingers<br>- gallstones in children<br>- biliary cirrhosis<br>- portal hypertension<br>- esophageal varices<br>- splenomegaly<br>- recurrent pancreatitis<br>- growth and weight deficiency<br>- recurrent swelling of the parotid glands<br>- delayed puberty<br>- male infertility (obstructive azoospermia)<br>- diabetes

The data presented in Table S1 are summarized from the literature cited in the main manuscript [8,12–16].
